# AI-assisted neurocognitive assessment protocol for older adults with psychiatric disorders

**DOI:** 10.3389/fpsyt.2024.1516065

**Published:** 2025-01-13

**Authors:** Diego D. Díaz-Guerra, Marena de la C. Hernández-Lugo, Yunier Broche-Pérez, Carlos Ramos-Galarza, Ernesto Iglesias-Serrano, Zoylen Fernández-Fleites

**Affiliations:** ^1^ Department of Psychology, Faculty of Social Sciences, Universidad Central “Marta Abreu” de Las Villas, Santa Clara, Villa Clara, Cuba; ^2^ Applied Behavior Analysis Department, Prisma Behavioral Center, Miami, FL, United States; ^3^ Centro de Investigación en Mecatrónica y Sistemas Interactivos - MIST, Facultad de Psicología, Universidad Tecnológica Indoamérica, Quito, Ecuador; ^4^ ”Dr. C. Luis San Juan Pérez” Provincial Teaching Psychiatric Hospital, Santa Clara, Villa Clara, Cuba

**Keywords:** neurocognitive assessments, neuropsychology, older adults, artificial intelligence, cognitive disorders

## Abstract

**Introduction:**

Evaluating neurocognitive functions and diagnosing psychiatric disorders in older adults is challenging due to the complexity of symptoms and individual differences. An innovative approach that combines the accuracy of artificial intelligence (AI) with the depth of neuropsychological assessments is needed.

**Objectives:**

This paper presents a novel protocol for AI-assisted neurocognitive assessment aimed at addressing the cognitive, emotional, and functional dimensions of older adults with psychiatric disorders. It also explores potential compensatory mechanisms.

**Methodology:**

The proposed protocol incorporates a comprehensive, personalized approach to neurocognitive evaluation. It integrates a series of standardized and validated psychometric tests with individualized interpretation tailored to the patient’s specific conditions. The protocol utilizes AI to enhance diagnostic accuracy by analyzing data from these tests and supplementing observations made by researchers.

**Anticipated results:**

The AI-assisted protocol offers several advantages, including a thorough and customized evaluation of neurocognitive functions. It employs machine learning algorithms to analyze test results, generating an individualized neurocognitive profile that highlights patterns and trends useful for clinical decision-making. The integration of AI allows for a deeper understanding of the patient’s cognitive and emotional state, as well as potential compensatory strategies.

**Conclusions:**

By integrating AI with neuro-psychological evaluation, this protocol aims to significantly improve the quality of neurocognitive assessments. It provides a more precise and individualized analysis, which has the potential to enhance clinical decision-making and overall patient care for older adults with psychiatric disorders.

## 1 Introduction

In the fields of neuropsychology and psychiatry, the neurocognitive assessment of older adults with psychiatric disorders presents both a significant challenge and a crucial area of study due to the complexity of mental and cognitive processes involved in this population (1, 2). The intersection between positivist neuropsychology and Luria’s qualitative neuropsychology emerges as a pivotal point in understanding and evaluating these individuals (3).

Positivist neuropsychology, rooted in scientific methods and objectivity, relies on standardized and validated psychometric tests, such as those adapted for the Cuban population. This approach provides a solid foundation for cognitive evaluation through quantitative and comparative measurements, helping to identify cognitive patterns and deficits with precision and reproducibility (4, 5).

Conversely, qualitative neuropsychology, inspired by Alexander Luria’s work, focuses on understanding the complexity and individuality of cognitive processes through a holistic and detailed approach. This qualitative perspective involves in-depth analysis of various dimensions of cognition and behavior, acknowledging the influence of contextual and personal factors on mental functions (3).

Alexander Luria’s neuropsychological approach is notable for its focus on the dynamic interactions between various brain functions and the significance of cultural and social contexts in studying the human mind (3). Luria proposed a triarchic model that categorizes brain functions into three main units. The First Unit includes structures in the brainstem, such as the reticular activation system, thalamus, and monoaminergic cell groups, which collaborate to activate and sustain the cortical tone needed to stimulate the cerebral cortex, thus fostering alertness. The Second Unit comprises the parietal, occipital, and temporal lobes, responsible for acquiring, processing, integrating, and storing sensory information from the environment. The Third Unit consists of the frontal lobe, which manages the selection, planning, execution, and direction of behavior patterns and their evaluation. It also includes crucial cognitive functions like sustained attention, awareness, and insight (6). Luria’s holistic approach has been vital in understanding the complexity of the human brain and its capacity for adaptation and change over time (7).

The convergence of these theoretical approaches within the neurocognitive assessment protocol for older adults with psychiatric disorders represents a significant advancement in the field. Combining the scientific rigor of positivist neuropsychology with the clinical sensitivity and personalized approach of qualitative neuropsychology results in a more comprehensive and profound evaluation of cognitive health in this population (8, 9).

Moreover, the use of artificial intelligence (AI) in analyzing results offers invaluable support to the assessment process. Through sophisticated algorithms and advanced computational capabilities, AI can detect subtle patterns, complex correlations, and significant trends in large datasets that may elude human detection (10, 11). This integration enables a coherent and efficient synthesis of objective and subjective data, minimizing interpretative and evaluative errors. AI’s ability to process vast amounts of data quickly and simultaneously allows for a more thorough and detailed assessment, revealing hidden connections and meanings that might be missed in manual analysis (12, 13).

Integrating AI into the neurocognitive assessment process is likely to create a synergy between machine analytical capabilities and clinical sensitivity. This results in a more comprehensive and accurate approach to understanding psychiatric disorders in older adults. This blend of technology and clinical expertise not only enhances diagnostic quality but also paves the way for more effective and personalized interventions, significantly improving the quality of life for these individuals. However, even though the background on the use of AI for diagnosis in mental health is varied, contemporary literature emphasizes that more research is needed to refine AI algorithms and improve their accuracy and applicability in clinical settings. This includes external validation of models and international collaboration to share data and knowledge (14). Overall, this protocol represents a significant advancement in assessing older adults with psychiatric disorders, combining scientific rigor, clinical sensitivity, and the analytical power of AI for a integrative and precise understanding of mental and cognitive health in this vulnerable population.

The main objective of this study is to present a comprehensive neurocognitive assessment protocol for older adults. Additionally, it aims to leverage artificial intelligence (AI) to analyze the assessment results, allowing for the detection of subtle patterns and correlations that may not be evident through traditional methods. Ultimately, our goal is to improve diagnostic accuracy and inform personalized interventions, thereby enhancing the quality of life for older adults facing the dual challenges of cognitive decline and psychiatric disorders.

## 2 Materials and equipment

This study will focus on the detailed presentation of a neurocognitive assessment protocol specifically designed for older adults with psychiatric disorders. It will integrate the principles of positivist neuropsychology with Luria’s qualitative neuropsychology (1, 15). By combining these approaches, the protocol aims to address both the quantitative and qualitative aspects of neurocognitive assessment for this particular population.

By integrating these complementary approaches, the protocol aims to offer a comprehensive assessment that accurately identifies cognitive deficits, taking into account the emotional and social context in which these disorders manifest in older adults with psychiatric disorders. This integrative analysis will not only improve the accuracy in identifying cognitive deficits but also provide a broader understanding of the factors influencing cognitive function in this population.

### 2.1 Target population

The target population for this protocol is older adults, who often face a decline in cognitive functions due to normal aging or conditions like dementia (16). This decline presents unique challenges for neurocognitive assessments, necessitating specialized approaches. Additionally, psychiatric disorders are prevalent among the elderly and can interact with cognitive decline in complex ways, making it essential to address this comorbidity. Many older adults may also face social isolation, economic challenges, and a lack of support systems, all of which can exacerbate cognitive decline and hinder their ability to seek help (17).

Moreover, many older adults have limited access to mental health services, which can delay the identification and treatment of cognitive and psychiatric issues. As the population ages, there is an increasing need to enhance neurocognitive assessments for this demographic (1). In Cuba, older adults often encounter specific social determinants of health that can influence their cognitive health. These include limited access to mental health services, which can delay the identification and treatment of cognitive and psychiatric issues. The healthcare system, while comprehensive in many respects, may not always adequately address the unique needs of the elderly, particularly in rural areas where resources are scarce. Additionally, cultural stigma surrounding mental health can further complicate access to care, leading to underdiagnoses and undertreatment of cognitive disorders (18). The proposed protocol seeks to meet this need innovatively by utilizing artificial intelligence, which can improve access and facilitate early detection of neurocognitive deficits in older adults (13).

### 2.2 Approach

For these reasons, the development of the neurocognitive assessment protocol for older adults with psychiatric disorders will involve a meticulous process of integration and adaptation of established and validated psychometric tests. This careful approach aims to ensure the relevance and accuracy of the assessment for this complex and vulnerable population.

When selecting assessment tools, special attention will be paid to the range of cognitive domains relevant to the target population. Key areas such as:

- Memory: Essential for daily functioning and quality of life in older adults.
- Attention: Often affected by various psychiatric disorders.
- Executive Functions: Crucial for planning and decision-making.
- Visuospatial Skills: Indicative of potential visual processing and spatial perception issues.

Additionally, other relevant cognitive functions such as language, perception, and social cognition will be considered, as these may be affected in older adults with psychiatric disorders. The careful selection of instruments that address these diverse cognitive areas will allow for a comprehensive and multidimensional evaluation of cognitive function in this specific population.

The protocol will take into account potential sensory, cognitive, or emotional limitations that may influence the validity of assessments. The selected tools will be chosen to be appropriate and effective for the target population, maximizing the reliability and validity of the results obtained.

### 2.3 Instrumentation

#### 2.3.1 Addenbrooke’s Cognitive Examination

The ACE-III is a widely used neuropsychological test consisting of five sections that assess different cognitive domains: orientation, memory, verbal fluency, language, and visuospatial skills. It is a valuable tool for detecting and monitoring cognitive disorders, such as mild cognitive impairment and dementia, providing a detailed and quantitative evaluation of cognitive function (19, 20).

#### 2.3.2 INECO frontal screening

The IFS is designed to specifically assess executive functions, which are higher-level cognitive abilities necessary for planning, organizing, problem-solving, maintaining attention, and controlling behavior. This assessment focuses on aspects such as cognitive flexibility, inhibition, and working memory, making it particularly relevant for older adults with psychiatric disorders who may struggle in these areas (21).

#### 2.3.3 Geriatric Depression Scale

The GDS is a widely used tool for evaluating the presence and severity of depressive symptoms in older adults. It consists of a series of simple and direct questions addressing emotional and affective aspects common in depression, allowing for the identification of potential depressive signs in this vulnerable population. Given that depression can significantly impact cognitive function, this scale is an important component of the assessment (22).

#### 2.3.4 Lawton & Brody Scale for instrumental activities of daily living

The LBI scale assesses an individual’s ability to perform complex and independent daily activities, such as managing finances, shopping, preparing meals, and using the telephone. This evaluation provides valuable information about the autonomy and functionality of older adults, aspects that can be affected by psychiatric disorders and are essential for assessing cognitive and functional status (23).

#### 2.3.5 Cognitive Reserve Scale

The Cognitive Reserve Scale evaluates the brain’s ability to withstand damage before clinical symptoms emerge. It considers factors such as education, cognitively stimulating activities throughout life, and occupational history, which may influence an individual’s ability to compensate for or mitigate the effects of potential cognitive disorders. We selected the scale proposed by León-Estrada et al. (24) which is the most suitable for assessing cognitive reserve in this specific context. The choice of this version is based on two main criteria. First, the scale records three comprehensive domains: activities of daily living (which includes the category “functional autonomy,” referring to the subject’s independence in performing basic daily activities), education-information (composed of the category “knowledge expansion,” which registers participation in activities related to education and the increase of knowledge and language skills, aspects that contribute to the plasticity associated with cognitive reserve), and hobbies-free time (which includes the categories “leisure activities” and “physical level”; in leisure activities, hobbies and interests that the subject engages in during their free time are included). The second criterion considered is its scoring system. This scale features a scoring system that includes measurements across three stages of life (Youth 18–35 years; Adulthood 36–64; Older Adulthood 65 years or older). In this way, it provides a dynamic estimate of reserve, not limited to the present, which allows exploration of the construct from a multifactorial and multitemporal dimension. This scale enables a precise comparison between past and present cognitive reserve, aligning with Luria’s historical-cultural approach, emphasizing the importance of understanding cognitive development within a temporal and cultural context. Assessing cognitive reserve is relevant for understanding cognitive adaptation in older adults with psychiatric disorders and for designing personalized intervention strategies (24, 25).

## 3 Methods

### 3.1 Artificial intelligence model design

In this study, an approach based on the use of prompts generated by OpenAI will be implemented, a free online system that relies on advanced machine learning language models. The prompts generated by OpenAI-InstructGPT will be created from a specialized natural language architecture, trained on an extensive corpus of textual data to generate coherent and contextually relevant responses. InstructGPT was selected as the artificial intelligence model due to its ability to understand and interpret natural language, which is essential for processing participants’ responses during assessments and interviews. This model can quickly analyze and interpret data, facilitating the identification of trends and relationships among the evaluated neurocognitive dimensions. Additionally, InstructGPT can generate responses based on input data and prompts, making it a valuable tool for creating reports based on assessment results (26–28).

The specific tasks expected of this artificial intelligence model in the context of cognitive assessment include understanding the scores from neurocognitive tests, clinical notes, and in-depth interviews. It is anticipated that the model’s linguistic analysis and contextualization of information gathered during interviews will enable it to generate insights and personalized recommendations for diagnosis, treatment, and follow-up care for participants. Additionally, the model is intended to provide tailored suggestions for interventions and management strategies based on individual needs.

Therefore, the artificial intelligence model to be implemented will be characterized by its ability to generate contextually appropriate and relevant responses based on the provided prompts. This will facilitate both content writing and exploration of specific topics within the domain of neurocognitive assessment conducted. By not relying on traditional programmatic code, the prompt-based approach of OpenAI-InstructGPT will offer flexibility and adaptability in response generation based on the needs and requirements of the study. The model will process the data through a structured workflow that includes the following stages (**Table 1**):

**Table 1:** Artificial intelligence process followed in the protocol.

Stages	Description
Data Reception	InstructGPT model will receive data in the form of prompts containing relevant information about the participants, including test scores, clinical notes, and interview transcripts.
Natural Language Analysis	The model will decompose and analyze the text to identify key concepts, language patterns, and semantic relationships. This analysis allows the model to understand the context and intent behind the participants’ responses.
Recommendation Generation	Based on the previous analysis, InstructGPT will generate personalized recommendations for the diagnosis, treatment, and follow-up of the participants. This will include specific suggestions for interventions and management strategies tailored to individual needs, using clear and accessible language.
Contextualization and Personalization	The model will contextualize the information gathered during the interviews, integrating data from different sources to provide a more comprehensive view of each participant’s cognitive and emotional state. This allows the recommendations to be more precise and relevant.
Report Generation	Finally, the model created based on InstructGPT will be able to compile all the analyzed information and generated recommendations into comprehensible reports that can be used by healthcare professionals to make informed decisions regarding patient care.

The artificial intelligence model was meticulously trained using prompts derived from a pilot study conducted with a sample of 15 patients, ranging in age from 65 to 82 years, who attended psychogeriatric consultations at the Psychiatric Hospital of Villa Clara, Cuba. During the pilot study, these patients were administered the standardized questionnaires mentioned earlier, and in-depth interviews were conducted to capture a wide range of cognitive and emotional data that were not fully elucidated in the questionnaires.

For the selection of the 15 patients, inclusion criteria were applied that considered diversity in terms of diagnosis, severity of symptoms, and treatment history. We used a purposive sampling method, aiming to include patients who represented different clinical profiles within the target population. While we recognize that 15 patients are a limited number, this pilot phase will allow us to make initial adjustments to the model and assess its feasibility before proceeding to a broader evaluation phase. Because of that, our intention is to carry out a second evaluation stage of the model, which will serve as a preliminary step before its official clinical application. In this phase, we plan to include patients who were not part of the initial sample, which will allow us to obtain a more comprehensive and representative view of the model’s effectiveness.

The information collected from this pilot study was used to train the artificial intelligence model, enabling it to learn and recognize complex patterns in neurocognitive assessment data. The model was trained using a combination of data, including scores from psychometric tests and demographic details of the 15 patients involved in the study. These data were integrated through a normalization and coding process to ensure consistency and quality in the training dataset.

To evaluate the model’s performance, accuracy and sensitivity analyses were conducted (see Formulas 1 and 2). The model achieved an accuracy of 73.33%, reflecting the proportion of correct predictions relative to the total number of predictions made. Additionally, a sensitivity of 90.90% was obtained, indicating the proportion of actual cases accurately identified by the model.


$$\begin{array}{c} Accuracy=\frac{True\;Positive+True\;Negative}{True\;Positive+True\;Negative+False\;Positive+False\;Negative}\\ =\frac{10+1}{10+3+1+1}=0.7333=73.33\% \end{array}$$

**Formula 1. Accuracy calculation**


$$\begin{array}{c} Sensitivity=\frac{True\;Positive}{True\;Positive+False\;Negative}=\frac{10}{10+1}\\ =0.9090=90.90\% \end{array}$$

**Formula 2. Sensitivity calculation**

Once the model was trained, it underwent an evaluation phase by a multidisciplinary committee composed of 9 experts in psychiatry, neuropsychology, and psychology. These experts carefully reviewed the data generated by the artificial intelligence model and agreed that the data provided a higher level of diagnostic rigor and validity to neurocognitive assessment. The experts conducted a thorough review of the outputs generated by the artificial intelligence model, including the accuracy and sensitivity data. They also compared the model’s outputs with previously made human judgments. For this comparison, the experts used a qualitative approach based on the review of clinical histories, which allowed them to analyze individual cases in depth and assess how the model’s conclusions aligned with the existing clinical information.

They highlighted the model’s ability to identify subtle patterns and significant correlations in the data, as well as its capacity to provide additional information that enriched the understanding of cognitive and emotional function in the evaluated patients.

### 3.2 Procedure and ethical considerations

To determine the number of participants for the study, a careful estimation of the sample size was conducted on G*Power (v.3.1.9.7), considering the research complexity, expected data variability, and the capacity to detect significant effects aligned with the study objectives. After thorough analysis, it was decided that the convenience sample will consist of 100 individuals.

Inclusion criteria were rigorously established to ensure the homogeneity and relevance of the sample. Participants must be aged between 65 and 85 years, diagnosed with specific psychiatric disorders, have attended psychogeriatric consultations within the last six months, and have provided informed consent to participate. Conversely, exclusion criteria were defined to prevent potential biases or interference in the results. Individuals with a history of degenerative neurological diseases, those undergoing intensive psychiatric treatments, or those with severe cognitive impairment will be excluded from the study.

Participant selection will be carried out randomly from the population that meets the inclusion criteria. Medical records and hospital databases will be utilized to identify potential participants. They will then be informed about the study and invited to participate voluntarily, ensuring confidentiality and respecting their autonomy in the decision-making process regarding their involvement in the research.

Evaluation process will involve individual assessments in clinical sessions conducted by neuropsychologists specialized in cognitive evaluation for older adults with psychiatric disorders. These sessions will be designed to provide a controlled and secure clinical environment, ensuring that participants can be assessed thoroughly and accurately, thereby maintaining the reliability and validity of the results.

In the design and execution of this study, fundamental ethical considerations established by the Declaration of Helsinki will be strictly adhered to. The protocol receive approval from the Institutional Review Board of “Dr. C. Luis San Juan Pérez” Provincial Teaching Psychiatric Hospital, Villa Clara, Cuba (2024-RX06F9 January 25, 2024). Fundamental ethical principles for research involving human subjects will be adhered to at all times. Confidentiality of the information collected during the evaluations will be strictly maintained, ensuring that participants’ personal data are protected and not disclosed without explicit consent. A rigorous informed consent process will be followed, where each participant will be clearly informed about the purpose of the evaluation, the procedures involved, potential risks and benefits, and their right to withdraw at any time without negative consequences. Respecting participants’ autonomy and dignity will be a priority throughout the evaluation process. These ethical practices will be crucial for ensuring the integrity of the research, protecting participants’ rights, and generating reliable and valid data that contribute to the advancement of knowledge in neuropsychology and geriatric psychiatry.

### 3.3 Data analysis

The results will be interpreted using a multimodal approach that combines both quantitative and qualitative analyses across different domains. The findings will be organized according to various evaluation areas, such as cognition, executive function, memory, and affectivity, among others. For each domain, a quantitative analysis will be performed, examining the standardized percentiles of each assessment technique.

In addition to the quantitative analysis, a qualitative analysis will be conducted, focusing on interpreting participants’ responses and performances in relation to their specific diagnostic profiles. This qualitative assessment will aim to contextualize the results within the framework of the differential diagnosis for each participant’s condition.

To enhance this interpretation, artificial intelligence (AI) analysis will be employed to identify patterns and complex relationships in the data that traditional methods might overlook. Utilizing machine learning techniques and data interpretation, AI will help uncover significant correlations between variables, explore the relationship between cognitive results and demographic and clinical variables, and reveal hidden patterns in the data that are not immediately apparent. This AI-driven analysis will provide a more detailed and accurate understanding of cognitive function and psychological well-being in older adults with psychiatric disorders.

Thus, the interpretation of the results will be based on a comprehensive and multidimensional understanding of the data, integrating the depth and richness of qualitative analysis, the precision and objectivity of quantitative analysis, and AI’s capability to identify intricate patterns and relationships. This synthesis of analytical approaches will allow for a more complete and detailed view of the findings, capturing the complexity of the phenomena studied, uncovering hidden relationships, and offering a deeper and more accurate understanding of cognitive function and psychological well-being in older adults with psychiatric disorders.

#### 3.3.1 Quantitative analysis

Quantitative data will be processed using SPSS software for each area of neurocognitive assessment addressed by the questionnaires. The analysis will focus on examining the standardized percentiles for each assessment technique used. Descriptive statistical measures, including means, standard deviations, and ranges, will also be calculated to provide a comprehensive overview of the quantitative results in each evaluated domain. Correlation tests and significance tests will be applied to explore relationships between quantitative variables and assess the significance of these relationships within the research context.

Additionally, the quantitative analysis will involve comparing participants’ results with established reference values, enabling a more precise contextualization and evaluation of each individual’s performance relative to the general population or specific reference groups.

#### 3.3.2 Qualitative analysis

The qualitative phase focused on the linguistic analysis of information obtained from participants. The research design employed is situated within the narrative constructivist paradigm. To support this analysis, we employ the six key steps proposed by Braun and Clarke (29). In-depth interviews served as the data collection technique, enabling the identification of various themes related to the participants’ cognitive states. All interviews were recorded and stored for subsequent analysis.

Once participant verbalizations were transcribed, an open coding process was conducted on the collected material. This involved analyzing the content of the interviews by comparing participants’ responses; similarities in ideas led to the emergence of qualitative categories, which are presented in the results. This process was further supported by the artificial intelligence program developed by the research team for diagnosing cognitive impairment.

#### 3.3.3 Artificial intelligence analysis

Quantitative data will be processed using SPSS software for each area of neurocognitive assessment addressed by the questionnaires. This analysis will be based on examining the standardized percentiles of each assessment technique used. Additionally, descriptive statistical measures such as means, standard deviations, and ranges will be calculated to provide an overview of the quantitative results in each evaluated domain. Correlation tests and significance tests will also be employed to explore relationships between quantitative variables and determine the importance of these relationships in the research context.

The quantitative analysis will also involve comparing the results obtained by Cubans participants with established reference values, allowing for a more precise contextualization and evaluation of each individual’s performance in relation to the general population or specific reference groups.

#### 3.3.4 Integrative analysis

The integration of quantitative results from psychometric tests and artificial intelligence analysis with qualitative findings has been thoughtfully planned. First, the quantitative results obtained from standardized psychometric tests and the analysis provided by artificial intelligence will be examined. This analysis aims to identify patterns, correlations, and significant trends in the data, offering an objective and structured foundation for understanding specific aspects of cognitive function.

Subsequently, qualitative findings from in-depth interviews and qualitative observations will be combined with the quantitative results and insights from artificial intelligence. By employing content analysis techniques and identifying emergent themes in the qualitative data, the experiences, perceptions, and emotions of participants regarding the tests and the neurocognitive assessment process will be explored. The correlation between the quantitative data, artificial intelligence findings, and qualitative insights will enable a more comprehensive understanding of the neurocognitive and emotional functioning of the study participants.

## 4 Anticipated results

**Figure 1:**
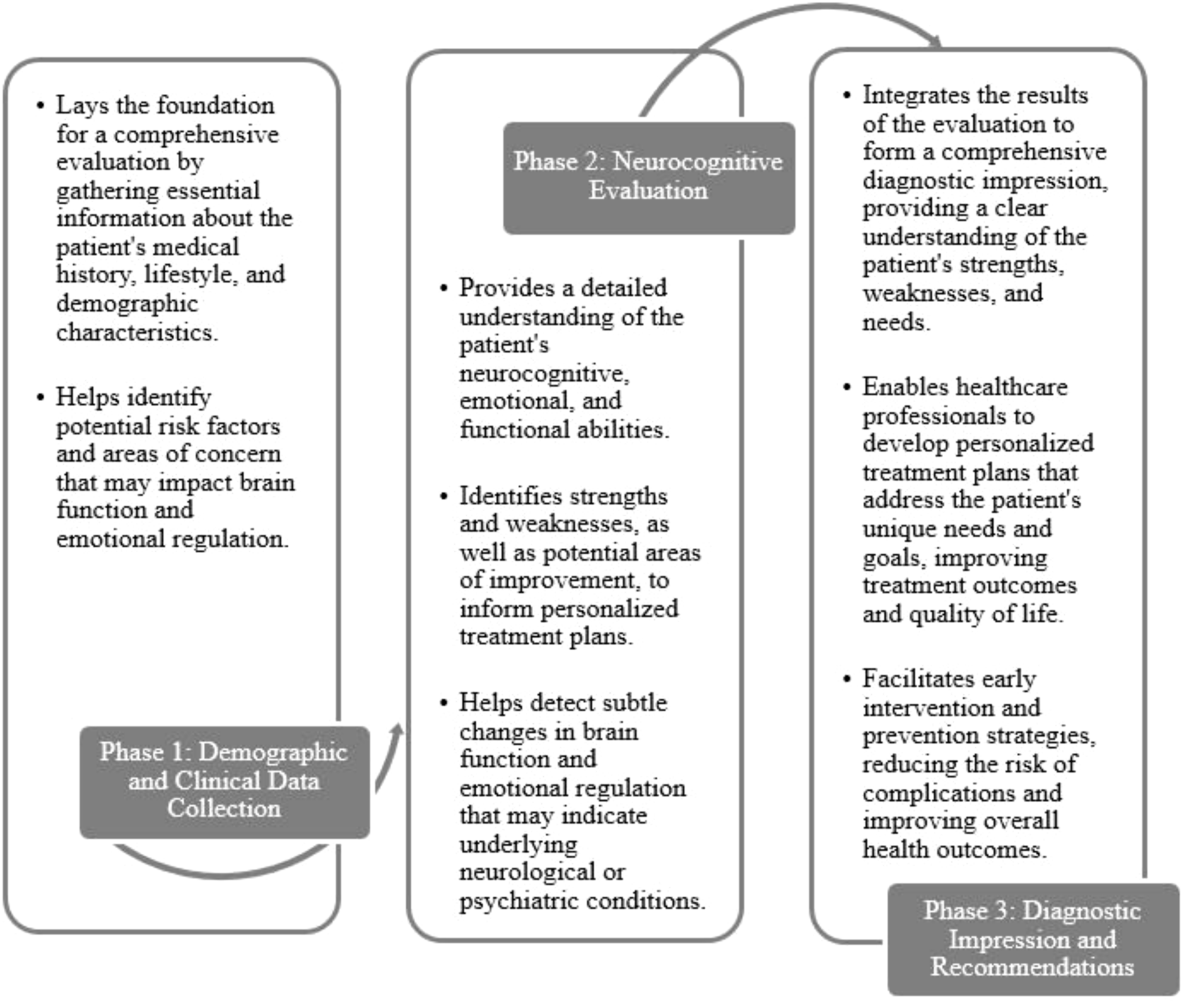
Phases of the proposed protocol.

The protocol presented in this study is structured into three essential phases (see **Figure 1**) and is notable for its ability to integrate the two fundamental approaches of neuro-psychology by employing standardized and validated psychometric tests tailored specifically for the Cuban population. However, the diversity within psychiatric populations studied presents a unique challenge, as the statistical adaptation of traditional techniques may be limited due to insufficient prevalence data to ensure representative samples.

To address this complexity, the evaluation is approached qualitatively, with an individualized focus that examines each dimension of the assessment in relation to the specific pathology of each patient. A comprehensive evaluation is conducted, considering not only the patient’s psychiatric condition but also their cognitive, emotional, and instrumental functioning, socio-demographics and cultural factors (age, gender, education, occupation, socioeconomics condition, lifestyles, supports networks), as well as the potential for compensation through cognitive reserve.

To enhance diagnostic accuracy and mitigate the impact of subjective biases in the assessment of older adults with psychiatric disorders, an innovative component has been integrated into the evaluation protocol: artificial intelligence (AI)-assisted analysis. This strategic addition aims not only to complement but to enrich and deepen neurocognitive diagnosis through a more objective and analytical perspective.

### 4.1 Phase 1: collection of demographic and clinical data

The collection of demographic and clinical data is a crucial step in the neurocognitive and emotional evaluation process (30). This phase aims to gather relevant information about the patient to understand their context and complexity (see **Table 2**). The information collected in this phase will serve as the foundation for the subsequent neurocognitive and emotional evaluation and will be essential for identifying patterns and trends that may influence the evaluation results.

**Table 2:** Patient sociodemographic variables.

Variable	Definition
Age	It is an important factor that can influence the patient’s cognitive and emotional function (31).
Gender	It can influence the presentation and progression of psychiatric disorders (32).
Education	It provides information about the patient’s level of education, which can influence their ability to understand and perform cognitive tasks (33).
Occupation	It provides information about the patient’s current or previous work activity, which can influence their cognitive and emotional functioning (34).
Handedness	It refers to the patient’s handedness, which can influence cognitive and emotional functioning (35).
Psychiatric history	They provide information about the patient’s history of psychiatric illnesses, which can influence the presentation and evolution of current symptoms (36).
Reason for Referral	It refers to the reason why the patient is seeking medical attention, which can influence the neurocognitive and emotional assessment (37).

The collection of these data is fundamental for establishing a solid foundation for the subsequent neurocognitive and emotional evaluation. Additionally, gathering this information allows for the identification of patterns and trends that may influence the evaluation results. Therefore, it is crucial that this phase is conducted thoroughly and accurately, as the information collected will guide the evaluation and diagnosis (38).

### 4.2 Phase 2: domain analysis

Once demographic and clinical data have been collected, the evaluation proceeds to domain-specific analysis. This phase involves four types of analysis: neurocognitive screening, emotional assessment, functional evaluation, and compensation possibilities. Additionally, diagnostic impressions and recommendations are formulated based on the results.

#### 4.2.1 Neurocognitive screening

In-depth neurocognitive screening employs standardized questionnaires such as the ACE-III (Addenbrooke’s Cognitive Examination III) and INECO-FS (INECO Frontal Screening) to assess the patient’s cognitive abilities. These tools are designed to evaluate the overall cognitive function of the patient, covering skills such as attention, memory, orientation, comprehension, and execution (19–21).

The advantage of using these tools is that they allow for a thorough and objective assessment of the patient’s cognitive function. Furthermore, incorporating artificial intelligence (AI) in the analysis of results helps identify patterns and complex relationships that may not be apparent through traditional methods. AI can analyze ACE-III and INECO-FS results in various ways, such as pattern recognition, identifying complex relationships, predicting outcomes, and personalizing care (13).

For example, AI can detect response patterns in memory tasks that indicate specific cognitive deficits. It can also uncover complex relationships between patient results and factors such as age, gender, or medical history (chronic diseases, previous neurological diseases, psychiatric disorders, medications, family history of neurodegenerative diseases, comorbidities). AI can predict future outcomes, such as the likelihood of cognitive decline or response to treatment. Moreover, AI can tailor medical care by recommending specific treatments and strategies to address cognitive and emotional deficits (12, 13, 39).

Thus, combining qualitative and quantitative methods with AI-based interpretations represents a critical advancement in neurocognitive evaluation. Utilizing these tools enables a comprehensive and objective assessment of cognitive function, leading to more personalized and effective medical care.

#### 4.2.2 Emotional assessment

Emotional assessment is a crucial component of neurocognitive evaluation. The patient’s emotional state can significantly impact cognitive function, making it essential to evaluate this aspect to gain a complete understanding of their health (40, 41). The Geriatric Depression Scale (GDS) is used to assess the patient’s emotional state, specifically focusing on the presence and severity of depressive symptoms in older adults.

The advantage of using the GDS is that it provides an objective and reliable measure of the patient’s emotional state. The GDS is a widely used and validated tool for assessing depression in older adults, allowing for the identification of depressive symptoms such as sadness, loss of interest in activities, sleep disturbances, and fatigue (22).

AI analysis further enhances the interpretation of emotional assessment results by detecting patterns and complex relationships that traditional methods might miss. For example, AI can identify response patterns on the GDS that suggest a higher risk of major depression in patients with a history of chronic illness (42). AI can also uncover complex relationships between emotional assessment results and other factors, such as cognitive function, physical health, and medical history. This information can be used to develop personalized intervention strategies to address depression and improve cognitive function in these patients (12).

#### 4.2.3 Functional assessment

Functional assessment is crucial for evaluating a patient’s ability to perform daily activities, such as eating, personal hygiene, dressing, mobility, communication, and medication management. The Lawton & Brody Scale is used to assess the patient’s functional capacity. This tool is designed to evaluate the patient’s ability to perform daily activities independently and safely (43).

The advantage of using this tool is that it allows for an objective and reliable evaluation of the patient’s functional capacity. The Lawton & Brody Scale is widely used and validated for assessing functional ability in patients with physical or cognitive disabilities. This assessment helps identify areas of strength and weakness in the patient’s functional capacity, which is essential for developing personalized and effective care plans.

Furthermore, the use of artificial intelligence (AI) in analyzing the results enables the identification of trends and complex relationships that may not be apparent through traditional methods. AI can identify patterns and trends that may indicate a risk of function-al decline in the patient. Additionally, AI can uncover complex relationships between functional assessment results and other factors, such as cognitive function, emotional state, and the patient’s medical history (13).

#### 4.2.4 Compensation possibilities

Evaluating compensation possibilities is vital for identifying strategies that can be used to improve the patient’s cognitive and emotional function. The cognitive reserve scale is employed to assess the patient’s ability to compensate for cognitive and emotional deficits (24, 25). This tool provides an objective and reliable measure of compensation capabilities.

Cognitive reserve is a well-established measure used to evaluate the ability to compensate in patients with cognitive impairments. Assessing compensation possibilities helps identify areas of strength and weakness in the patient’s compensatory ability, which is crucial for developing personalized and effective care plans (44).

AI can further enhance this evaluation by identifying or corroborating data that may indicate a risk of cognitive or emotional decline. AI can also detect complex relationships between compensation assessment results and other factors, such as cognitive function, emotional state, and medical history. This information is valuable for addressing compensation capabilities and improving cognitive function in these patients (13).

### 4.3 Phase 3: diagnostic impression and recommendations

After completing the four evaluations (cognitive, emotional, functional, and compensation possibilities), the next step is to formulate the diagnostic impression and recommendations. The diagnostic impression is based on the patient’s performance on the questionnaires and differential diagnosis of the patient’s condition.

AI plays a crucial role in the diagnostic impression process. AI can assist healthcare professionals in making a more accurate and differential diagnosis by identifying key features of the patient’s symptoms and excluding other conditions that may present similar symptoms. Additionally, AI can provide an objective, evidence-based second opinion, which is especially valuable in cases of complex or rare conditions where diagnosis may be challenging (45, 46).

Once the diagnostic impression is established, individualized recommendations are provided based on the specific characteristics of the patient’s symptoms and needs. AI helps healthcare professionals develop more precise and personalized recommendations by identifying the most effective interventions for addressing the patient’s deficits and symptoms (47). Recommendations may include intervention strategies for cognitive and emotional deficits, as well as suggestions for improving functional ability and quality of life. AI can also help identify necessary resources and services and provide recommendations for accessing them (48).

AI is essential for individualizing treatment and providing personalized recommendations for each patient. It can also help reduce the time and resources needed to develop a treatment plan, improving the efficiency and effectiveness of the treatment. Ultimately, AI can enhance the patient’s quality of life by providing precise and personalized recommendations tailored to their specific needs and characteristics (47–49).

## 5 Discussion

The neurocognitive assessment protocol presented in this article offers a comprehensive and integrative tool for evaluating patients with psychiatric disorders. The combination of demographic and clinical data collection with domain-specific analysis provides a detailed and thorough understanding of the patient’s cognitive, emotional, functional, and compensatory abilities (38).

The use of standardized and validated instruments ensures the reliability and consistency of the neurocognitive assessment results (50). Additionally, the inclusion of compensation assessment and diagnostic impressions supported by artificial intelligence enhances the precision and objectivity of patient diagnosis and treatment (51, 52). Detailed diagnostic impressions and personalized recommendations based on the most effective strategies to address the patient’s specific deficits and symptoms enable early and effective interventions to improve the patient’s cognitive, emotional, and functional functions (48).

Moreover, the role of artificial intelligence in data analysis and diagnosis is crucial. AI enables greater accuracy and reliability in diagnosis, which can positively impact the patient’s quality of life. The use of AI in diagnostic impressions provides a more objective and precise evaluation of results, reducing subjectivity and variability in result interpretation. This is particularly important in neurocognitive assessment, where interpretation can be influenced by factors such as the healthcare professional’s experience and training (10, 12, 13).

Another important aspect is the protocol’s ability to identify patterns and complex relationships among various evaluated variables. This allows for a deeper understanding of the patient’s cognitive, emotional, and functional functions, which can help pinpoint specific areas requiring intervention. It is important to emphasize that neurocognitive assessment is not an isolated process but is integrated with clinical evaluation and patient treatment (44). The use of this protocol can be beneficial for healthcare professionals seeking to evaluate and treat patients with psychiatric disorders effectively and personally.

Regarding the protocol’s limitations, it is acknowledged that neurocognitive assessment is only one part of the comprehensive patient evaluation. Other factors, such as the patient’s medical history and laboratory test results, should also be considered when developing a treatment plan. Additionally, implementing the protocol may require increased training and resources for healthcare professionals, as well as the need for enhanced infrastructure and technology for data analysis and processing.

The use of artificial intelligence for neurocognitive assessment in conjunction with mental disorders such as major depression, bipolar disorder, and schizophrenia presents significant challenges due to the complexity and variability of symptoms. These disorders encompass a wide range of emotional, cognitive, and functional manifestations, making it difficult for algorithms to identify consistent patterns. Additionally, AI currently struggles to adequately integrate the personal, social, and cultural contexts that influence the development of these disorders, which limits its ability to provide accurate diagnoses with-out human review and oversight (53).

Another significant challenge AI faces is its inability to capture essential qualitative and subjective aspects in the evaluation of psychological symptoms. The diagnostic process requires not only the identification of quantifiable symptoms but also the interpretation of emotional and relational factors that emerge through human interactions. The lack of intuitive understanding in AI makes it insufficient to replace the necessary clinical judgment, especially in cases where the therapeutic relationship and patient context are crucial for accurate assessment. Therefore, its use is always recommended alongside human judgment (54).

One of the research team’s objectives is to apply the protocol in various sociodemographic, cultural, and linguistic contexts. Therefore, in the next phases of research, the protocol will be applied in various countries across Latin America and English-speaking countries. Additionally, there are populations in Latin America that do not have access to neuropsychological assessments to identify their cognitive level. Thus, another objective of the team is to bring the neurocognitive evaluation protocol to older adult populations who do not receive medical attention and need to identify their cognitive level and cognitive stimulation processes to reduce the impact of cognitive decline on their daily lives.

To achieve a generalization of the data processed by the protocol in neurocognitive evaluation, it is necessary to consider factors that determine the mental level of older adults, such as cognitive reserve generated by several factors, (for example, educational levels) (55). In this regard, a subsequent goal in this research process is to create norms according to the educational levels of the older adults who will be assessed in future studies. For example, we aim to generate normative parameters based on educational levels in the different countries where the protocol will be applied.

Artificial intelligence, despite its advancements, presents significant limitations. One of these is its difficulty in capturing the qualitative nuances of human language, such as irony or sarcasm (56). Additionally, AI models can reflect the biases present in the data they are trained on, which can lead to discriminatory outcomes (57). To mitigate these issues, it is crucial to diversify development teams, use high-quality and bias-free data, implement bias mitigation techniques, and promote model transparency and explainability. It is essential to recognize that AI is a powerful but imperfect tool. By proactively addressing its limitations, we can develop fairer, more equitable, and beneficial AI systems for society. This requires a multidisciplinary approach that combines technology with social sciences and humanities.

The integration of artificial intelligence in the healthcare field promises to revolutionize medical practice. Imagine a future where healthcare professionals have AI tools that allow them to analyze large volumes of medical data quickly and accurately, thus supporting more informed clinical decision-making. However, this transition will not be without challenges. It will be necessary to train physicians to use these new tools effectively and understand their limitations. Additionally, significant investment in technological infrastructure will be required to ensure the compatibility of AI systems with existing electronic medical records (47, 58, 59).

One of the greatest challenges posed by AI in healthcare is its ability to handle complex cases. Neurodegenerative diseases such as Alzheimer’s or Parkinson’s, which often present overlapping symptoms, can be difficult to diagnose even for the most experienced physicians. AI algorithms are still in development and may struggle to differentiate between patterns of various diseases, especially when comorbidities are present (60, 61). To address this issue, it will be necessary to develop more sophisticated AI models that can analyze complex information and provide more accurate diagnoses.

Finally, it is essential to consider the ethical implications of AI in healthcare. Biases present in the data used to train algorithms can lead to discriminatory outcomes in medical care. For example, a diagnostic algorithm might underestimate the risk of certain diseases in specific population groups (57, 62). To avoid these problems, it is crucial to ensure that the datasets used to train algorithms are diverse and representative of the general population. Additionally, mechanisms of transparency and accountability must be implemented to ensure that AI systems are fair and equitable (63).

Finally, the use of artificial intelligence in this field raises ethical and privacy concerns, as the automated analysis of personal and sensitive data can pose security risks. Further-more, algorithms may be biased depending on the datasets used for training, which could impact diagnostic fairness across different demographic groups (64, 65). For these reasons, artificial intelligence should be viewed as a supplementary tool that supports, but does not re-place, the clinical expertise of mental health professionals.

### 5.1 Advantages and clinical implications

**Figure 2:**
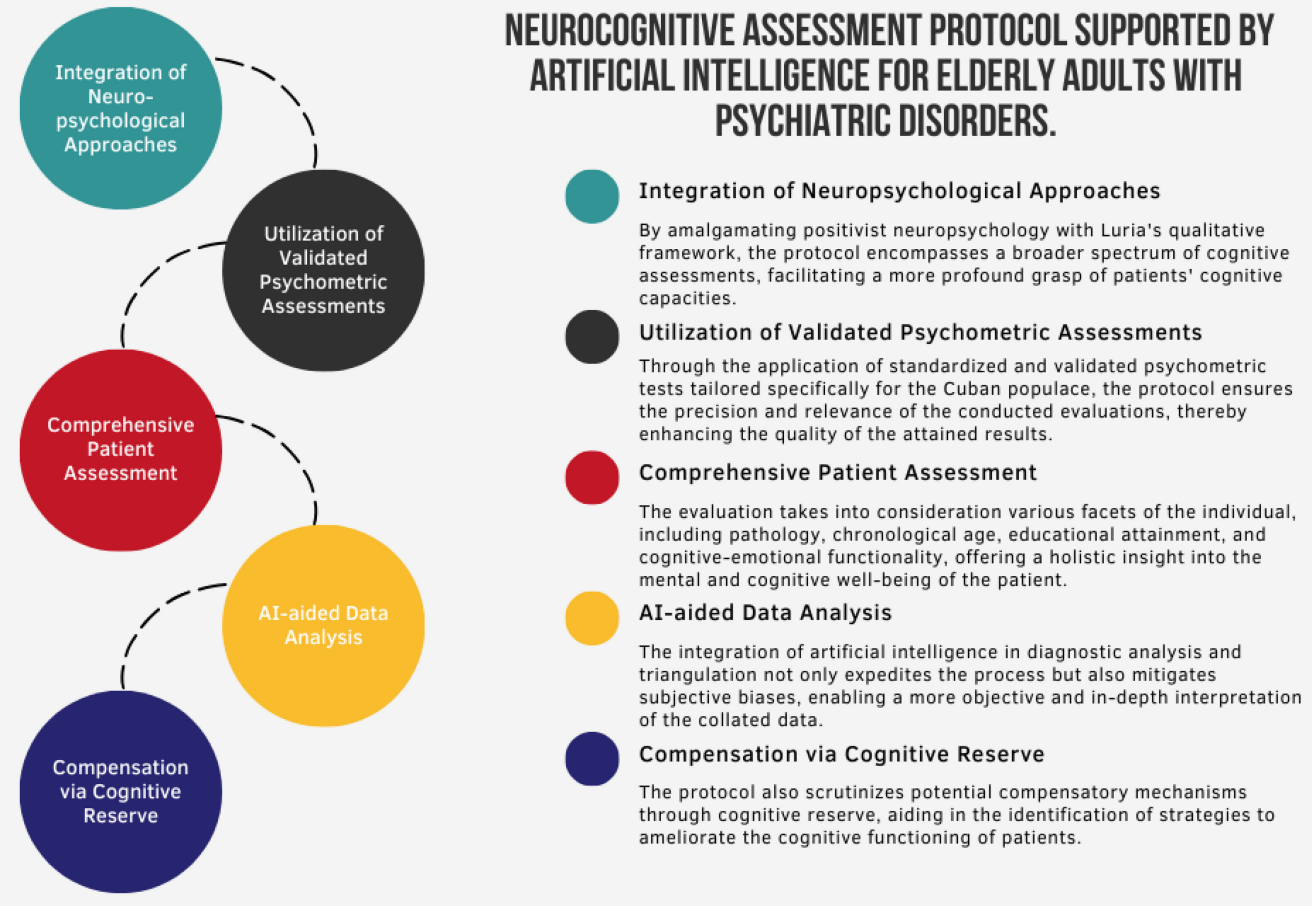
Description of the neurocognitive evaluation protocol.

The neurocognitive evaluation protocol has significant clinical implications and ad-vantages for medical practice (see **Figure 2**). Notably, it allows healthcare professionals to assess the cognitive, emotional, and functional function of patients with psychiatric dis-orders more precisely and objectively (see **Table 3**), which can help in developing more effective and personalized treatment plans.

**Table 3:** AI model´s clinical proposes for different psychiatric disorders.

Disorder (DSM-5)	Cognitive Impairments	Emotional Impairments	Functional Impairments	AI Adjustments	Assessment Instruments
Schizophrenia	Executive dysfunction, impaired working memory, attention deficits (55, 66)	Flat affect, emotional dysregulation (67)	Challenges in employment, self-care, and social isolation (68)	Analyze cognitive patterns; evaluate cognitive reserve’s role in recovery	INECO Frontal Screening, Addenbrooke’s Cognitive Examination III, Cognitive Reserve Scale
Alzheimer’s Disease	Progressive memory loss, impaired executive function, language difficulties (69)	Emotional distress, depression, anxiety (70)	Difficulty with daily activities, social withdrawal (71)	Monitor cognitive decline over time; assess cognitive reserve to mitigate decline	Addenbrooke’s Cognitive Examination III, Cognitive Reserve Scale, Geriatric Depression Scale
Frontotemporal Dementia	Personality changes, impaired social cognition, language deficits (72)	Apathy, emotional blunting, inappropriate emotional responses, facial emotion recognition impairments (73, 74)	Challenges in social interactions, managing finances, and taking medication (71, 75)	Analyze social cognitive patterns; evaluate cognitive reserve to guide care strategies	Addenbrooke’s Cognitive Examination III, Cognitive Reserve Scale
Vascular Dementia	Impaired attention, executive dysfunction, memory issues (76)	Emotional lability, depression (77)	Difficulty with planning, organizing, and carrying out tasks (71)	Monitor cognitive changes related to vascular health; assess cognitive reserve for intervention strategies	Addenbrooke’s Cognitive Examination III, Cognitive Reserve Scale, Geriatric Depression Scale
Generalized Anxiety Disorder	Difficulty concentrating, indecisiveness (78)	Excessive worry, irritability, and restlessness (79)	Impaired focus, avoidance of social situations (80)	Use predictive analytics to assess impacts on cognition; evaluate cognitive reserve as a buffer	Addenbrooke’s Cognitive Examination III, Geriatric Depression Scale, Cognitive Reserve Scale
Obsessive-Compulsive Disorder	Impaired focus due to intrusive thoughts (81)	Anxiety related to compulsions (82)	Time-consuming rituals affecting productivity (83)	Adjust task difficulty based on anxiety levels; assess cognitive reserve to understand coping strategies	Addenbrooke’s Cognitive Examination III, INECO Frontal Screening, Cognitive Reserve Scale
Post-Traumatic Stress Disorder	Memory impairments, concentration difficulties (84)	Hyperarousal, emotional numbing, re-experiencing symptoms (85)	Impaired functioning due to avoidance, and social withdrawal (86)	Analyze trauma-related cognitive deficits; evaluate cognitive reserve to enhance resilience	Addenbrooke’s Cognitive Examination III, Geriatric Depression Scale, Cognitive Reserve Scale
Borderline Personality Disorder	Impulsivity affects decision-making, and cognitive distortions (87)	Intense emotional fluctuations, and fear of abandonment (88)	Relationship crises, unstable job performance (89)	Detect patterns of emotional dysregulation; evaluate cognitive reserve for managing stress	Addenbrooke’s Cognitive Examination III, Geriatric Depression Scale, Cognitive Reserve Scale

### 5.2 Cost and resource implications

The psychometric tests employed in this study are freely available to the academic community, significantly lowering the initial costs of acquiring assessment tools. Additionally, the artificial intelligence model, trained using prompts, has been implemented with the open-source software OpenAI-InstructGPT, ensuring transparency and reducing expenses related to the development and execution of this essential component of the study. Standard computers are sufficient to carry out the various stages of the study protocol, which requires only basic infrastructure commonly found in academic and research settings.

Furthermore, after obtaining and analyzing the results from the protocol implementation, plans are in place to create and conduct training courses aimed at facilitating the successful application of this approach in other institutions. These courses will offer practical guidance on the protocol’s use while also promoting the dissemination and replicability of findings in a wider context.

## 6 Conclusions

This neurocognitive evaluation protocol is a valuable tool for assessing the cognitive and emotional functioning of patients with psychiatric disorders. The combination of neuropsychological approaches, validated psychometric tests, and artificial intelligence assisted analysis has been shown to enhance diagnostic accuracy and understanding of patients’ cognitive and emotional capacities. Despite challenges in statistical adaptation for diverse psychiatric populations, the protocol proves clinically relevant by offering a personalized approach that addresses the individual needs of each patient.

The practical applicability of the protocol is highlighted by the inclusion of artificial intelligence, which facilitates an objective analysis of data and reduces biases in result interpretation. This innovative approach holds significant potential for improving care quality in the field of geriatric psychiatry and opens new perspectives in neurocognitive assessment. This protocol not only stands out for its comprehensive approach and clinical relevance but also for its ability to combine traditional methods with advanced technology to provide more precise and relevant results in diagnosing older adults with psychiatric disorders. Ultimately, this neurocognitive evaluation protocol is a powerful tool for healthcare professionals seeking to assess patients with psychiatric disorders effectively and personally.

Finally, in this article, the research team’s interest has been to describe this protocol for evaluating cognitive impairments in older adults with psychiatric disorders. The next phase of research aims to apply the protocol to a significant sample to determine normative statistical values and conduct the respective reliability and validity analysis processes. Additionally, we will apply the protocol to a large number of older adults in Cuba, the United States, and Ecuador, so that we can gain a broader perspective on the technological proposal we are developing.

## Data Availability

The raw data supporting the conclusions of this article will be made available by the authors, without undue reservation.
